# The Training of Teachers

**Published:** 1903-09-12

**Authors:** 


### The Training of Teachers.

Education, with its numerous complications, is certainly one of the questions of the hour. There is much shouting of the captains and marshalling of rival hosts. The noise of the conflict is in all our ears. With the merits of the immediate quarrel we have here no concern. All, however, may regard it as so much to the good that the attention of the nation should be turned to the question of Education, even at the cost of public controversy.

There is little necessity to emphasize the importance of educational issues in the constituency to which we speak. Medical men have necessarily had a special educational experience, and part of this has been enjoyed at an age when the mind is fairly competent both to appraise merits and to detect defects in the system of training. These considerations suggest a special claim for attention to the recently issued Report on Training Colleges by Mr. A. Rankine, one of His Majesty's Chief Inspectors. We cannot pretend to discuss the details of this Report. Our object is rather to emphasise its argument that the thorough training of teachers in the principles and art of their profession lies at the very foundation of educational success. How far this is from being duly appreciated, either by the public, or by those who hold the patronage of teaching appointments, is a matter of common experience. Mr. Rankine's Report tells us that on the staffs of the elementary schools there are at the present day 28,000 teachers who have never passed through a college; that the present training colleges cannot accommodate one half of those who try to gain admission; and that this inadequate accommodation is the consequence of too scanty grants of public money. Evidently, whilst the theory that those who have failed in other directions are at least fitted to train and develop the minds of youth no longer receives general countenance, the enormous national importance of the schoolmaster is far from being widely recognised. He is allowed to be a necessity, but there are few among the crowd who appreciate his real value in the body politic. "One green field is the same as every other green field," said Dr. Johnson; and that is very much the view which the British public takes of its schoolmasters.

A similar policy is largely adopted in specialised and technical education. The merits of the teacher, as such, receive for the most part but scant consideration at the hands of those who control the nominations to the staffs of our universities, colleges, and medical schools. Instances are notorious of men selected for teaching work, not only devoid of the essential qualities of a teacher, but on the basis of a candidature innocent of all credentials suggesting any special ability in this direction. The patrons, it is only too evident, place such credentials low down in the scale of merit. What is the consequence? Inevitably, that those who entertain ambitions towards these apppointments spend their time and energy in the cultivation of qualities which are more likely "to pay" than is efficiency in the teaching art. The bad tradition is thus perpetuated, and teachers who cannot teach continue and abound. Probably the really great teacher is born rather than made. But every encouragement should be given to the development and culture of moderate talents. For the man who cannot or will not learn, the verdict should be: Neither shall he teach. It is sometimes said—and there are well-known examples which appear to confirm the statement—that many men of great capacity as original investigators, and in other directions, have no gift for teaching. In so far as this is true the answer is obvious—let such men keep to their special realm and leave teaching to those better fitted for it. But we are convinced that it would be found to be much less generally true were the principle once recognised that to gain a teaching appointment proficiency as a teacher is a necessity. As it is, custom, in this department of human work, saves incapacity from its natural penalty, and year after year permits the interests of students to be sacrificed on the altar of professorial dignity and inefficiency. The evil is of still wider extent. The whole value of teaching suffers depreciation in the general estimation. It becomes a common assumption that so far as teaching is concerned it does not much matter who is made responsible so long as books and opportunities for practical work exist. This we believe to be a serious mistake. The whole bent and direction of the mind in the elastic and flexible stage of youth is far more susceptible to the sway of personal influence than it is to any other force. National destiny lies with those who make our schoolmasters rather than with those who make our laws. As this truth gains recognition our training colleges for teachers will cease to be starved for lack of funds, and public opinion will pay to the efficient teacher the tribute which is his due.

